# Effects of Recombinant IL-13 Treatment on Gut Microbiota Composition and Functional Recovery after Hemisection Spinal Cord Injury in Mice

**DOI:** 10.3390/nu15194184

**Published:** 2023-09-27

**Authors:** Ibrahim Hamad, Jana Van Broeckhoven, Alessio Cardilli, Niels Hellings, Till Strowig, Stefanie Lemmens, Sven Hendrix, Markus Kleinewietfeld

**Affiliations:** 1 VIB Laboratory of Translational Immunomodulation, Center for Inflammation Research (IRC), Hasselt University, 3590 Diepenbeek, Belgiumalessio.cardilli@uhasselt.be (A.C.); 2 Department of Immunology and Infection, Biomedical Research Institute (BIOMED), Hasselt University, 3590 Diepenbeek, Belgium; jana.vanbroeckhoven@uhasselt.be (J.V.B.); niels.hellings@uhasselt.be (N.H.);; 3 Department of Microbial Immune Regulation, Helmholtz Center for Infection Research, 38124 Braunschweig, Germany; 4 Institute for Translational Medicine, Medical School Hamburg, 20457 Hamburg, Germany

**Keywords:** spinal cord injury, inflammation, microbiome, dysbiosis, interleukin-13, regeneration

## Abstract

In recent years, the gut–central nervous system axis has emerged as a key factor in the pathophysiology of spinal cord injury (SCI). Interleukin-13 (IL-13) has been shown to have anti-inflammatory and neuroprotective effects in SCI. The aim of this study was to investigate the changes in microbiota composition after hemisection injury and to determine whether systemic recombinant (r)IL-13 treatment could alter the gut microbiome, indirectly promoting functional recovery. The gut microbiota composition was determined by 16S rRNA gene sequencing, and correlations between gut microbiota alterations and functional recovery were assessed. Our results showed that there were no changes in alpha diversity between the groups before and after SCI, while PERMANOVA analysis for beta diversity showed significant differences in fecal microbial communities. Phylogenetic classification of bacterial families revealed a lower abundance of the Bacteroidales S24-7 group and a higher abundance of Lachnospiraceae and Lactobacillaceae in the post-SCI group. Systemic rIL-13 treatment improved functional recovery 28 days post-injury and microbiota analysis revealed increased relative abundance of Clostridiales vadin BB60 and *Acetitomaculum* and decreased *Anaeroplasma*, *Ruminiclostridium_6*, and *Ruminococcus* compared to controls. Functional assessment with PICRUSt showed that genes related to glyoxylate cycle and palmitoleate biosynthesis-I were the predominant signatures in the rIL-13-treated group, whereas sulfolactate degradation super pathway and formaldehyde assimilation-I were enriched in controls. In conclusion, our results indicate that rIL-13 treatment promotes changes in gut microbial communities and may thereby contribute indirectly to the improvement of functional recovery in mice, possibly having important implications for the development of novel treatment options for SCI.

## 1. Introduction

In recent years, the interplay between the gut microbial network and the central nervous system (CNS, Supplementary Table S1) has attracted considerable interest [1,2]. The proper functioning of this network is important for the development of the CNS, but it is also considered a crucial factor in the functional outcome of various neurological disorders [3,4,5]. Recent research has shown that there is a bidirectional relationship between the CNS and the gut microbiota [6]. Perturbations in the CNS lead to changes in the luminal/mucosal habitat of the gut, which in turn may affect microbial composition [7,8]. On the other hand, disruptions in the gut microbiota have been shown to contribute to a variety of neurologic diseases, including multiple sclerosis (MS), Parkinson’s disease, and autism spectrum disorder (ASD) [3,4,5]. Studies indicate that MS is associated with a dysbiosis of the gut microbiota [3,9]. Alterations in the gut microbiome of MS patients may include an increase in proinflammatory and neurotoxic species and a decrease in bacterial species that produce immune tolerance-promoting molecules such as short-chain fatty acids (SCFAs) [3,10]. In addition, several studies have shown that in Parkinson’s disease, there is an increase in the bacteria *Lactobacillus*, *Akkermansia*, and *Bifidobacterium* and a decrease in bacteria from the Lachnospiraceae family [11,12,13,14]. These changes are thought to contribute to increased gut permeability and systemic inflammation that may exacerbate neuroinflammation and neurodegeneration [4]. Similarly, changes in the composition of the gut microbiota have been observed in individuals with ASD [5,15]. Studies have found lower levels of beneficial bacteria such as *Bifidobacterium* and higher levels of *Lactobacillus*, *Clostridium*, Bacteroidetes, and *Desulfovibrio* [15]. These imbalances may contribute to the gastrointestinal (GI) symptoms commonly observed in people with ASD and may also affect their neurological symptoms [15].

Spinal cord injury (SCI) is a devastating condition that results in the disruption of motor, sensory, and autonomic functions below the level of injury. Each year, approximately 250,000–500,000 people suffer from an SCI [16,17,18,19]. Most SCI cases are caused by trauma, e.g., car accidents, falls during sports, etc., although in some cases, it may have a nontraumatic origin, such as a tumor or infection. The pathophysiology of SCI is biphasic. First, the initial mechanical injury leads to excessive cell death, destroyed vascular structures, hemorrhage, etc. This, in turn, triggers a complex secondary injury phase characterized mainly by a strong inflammatory response and the formation of a glial scar, both of which impede spontaneous regeneration at later stages [20]. Due to the multifactorial nature of SCI pathophysiology, the development of therapies is a major challenge. Currently, the golden standard for treating SCI patients is spinal cord decompression, corticosteroid administration, and physical rehabilitation [21,22]. In addition, because SCI patients are at high risk for obesity and various chronic cardiometabolic disorders, it is suggested that these individuals maintain a healthy lifestyle/nutrition [23]. Besides paralysis, patients with SCI, suffer from secondary comorbidities that significantly affect their quality of life [24,25,26]. These include GI dysfunction, which leads to altered gastric emptying and shortening of the GI transition period [27,28]. They are caused by a loss of autonomic control of the stomach due to a disturbed axis between the intestine and the CNS [24,29,30,31,32,33,34]. In addition, SCI patients often have to undergo antibiotic treatment in response to recurrent infections, causing an extra challenge to their intestinal flora [35,36]. These GI problems are considered a major problem as they lead to hospitalization in up to 11% of cases [30,37,38]. Cumulative evidence showed an association between SCI and the gut microbiota. SCI results in the development of gut dysbiosis and reduced commensal abundance, which can lead to inflammation and other harmful effects [26]. Previous research has indicated that especially butyrate-producing bacteria are depleted after human and preclinical SCI [24,33,39,40]. In addition, there is an increased production of pro-inflammatory cytokines such as TNF-α, IL-β, IL-6 and IFN-γ by activated immune cells and colon tissue, which contributes to the induction of pro-inflammatory macrophages that drive neuroinflammation after SCI [41,42]. Therefore, inducing a beneficial anti-inflammatory macrophage phenotype is desirable after SCI. We have previously demonstrated the importance of interleukin (IL)-13 on macrophage polarization towards an anti-inflammatory phenotype after SCI [43,44]. One of the key mechanisms by which IL-13 exerts its neuroprotective effect on SCI is the modulation of macrophage polarization [43]. In addition, IL-13 was found to influence the composition of the gut microbiota [45]. IL-13-overexpressing transgenic mice led to alterations in the composition of gut microbiota, characterized by a decreased abundance of *Lactobacillus*, *Bifidobacterium*, and *Anaerotruncus*, and increased abundance of *Emergencia*. These changes in the gut microbiota were associated with increased susceptibility to experimental colitis in mice [45].

To date, most research regarding microbiota alterations after SCI is performed in contusion and compression models characterized by the disadvantage of undamaged fibers that may lead to artifacts [46,47]. To avoid these problems, we first investigated changes in gut microbial composition in a hemisection SCI model characterized by the absence of uninjured fibers. In addition, we further investigated with this study the effects of anti-inflammatory recombinant (r)IL-13 treatment in SCI on microbiome composition and locomotion to identify changes in the gut microbiome that were correlated to functional recovery. 

To summarize, we found that hemisection SCI disturbs the homeostatic microbiota composition. Furthermore, rIL-13 treatment improved functional recovery after 28 days, and this was correlated with an enrichment in the presence of the Clostridiales vadin BB60. In addition, metabolic pathway analysis showed an increasing activity of the palmitoleate biosynthesis after rIL-13 treatment, suggesting an anti-inflammatory effect. 

## 2. Material and Methods

### 2.1. Animals 

Experiments were performed using female 10–12 week-old wild-type (WT) BALB/cJRj mice (Janvier Labs, Le Genest-Saint-Isle, France). Mice were housed in groups at the conventional animal facility of Hasselt University under standardized conditions (e.g., 12 h light/dark cycle, temperature-controlled room, food, and water ad libitum). Experiments were approved by the local ethical committee of Hasselt University (ethical ID 201968, 2 December 2019), and were performed according to the guidelines of the European Directive 2010/63/EU on the protection of animals for scientific purposes. 

### 2.2. T-Cut Hemisection Injury

A T-cut hemisection injury was performed as previously described [43,48,49]. In brief, mice were anesthetized with 2–3% isoflurane (IsofFlo, Abbot Animal Health, Wavre, Belgium) and were subjected to a partial laminectomy at thoracic level 8. Using iridectomy scissors, the left and right dorsal funiculi, the dorsal horns, and the ventral funiculus were transected, which resulted in a complete transection of the dorsomedial and ventral corticospinal tract. Afterward, the back muscles were sutured, and the skin was closed using wound clips (Autoclip, Clay-Adams Co., Inc., Becton-Dickinson, Erembodegem, Belgium). Post-operative care included (1) administration of a glucose solution (20%) to compensate for blood loss during surgery and (2) subcutaneous administration of buprenorphine (0.1 mg/kg, Temgesic, Val d’Hony Verdifarm, Beringe, Belgium) for pain relief. Of note, no antibiotics were given before or after surgery. Mice were placed in a temperature-controlled (33 °C) recovery chamber after surgery until they regained consciousness. Bladders were manually voided daily until 1-week post-injury followed by every other day until mice had a restored micturition reflex. Food intake was not measured during the experiment, but all animals received the same standard rodent chow throughout the entire study without any dietary supplements. 

### 2.3. Treatment

In total, 10 mice per group (either vehicle or treatment) were randomly operated on. Starting 1 h after surgery, mice received vehicle (PBS, Lonza, Verviers, Belgium) or rIL-13 (0.5 ug, Peprotech) once a day via intraperitoneal injection (i.p.) for 7 consecutive days. To prevent gut microbial cross-contamination between groups, animals receiving the same treatment were housed together. 

### 2.4. Locomotor Function Analysis

To assess functional recovery after SCI, the standardized Basso Mouse Scale (BMS) for locomotion was used [50]. The BMS is a 10-point rating scale based on hind limb movements in an open field, with a score 0 for complete hind limb paralysis, whereas a score 9 represents normal motor function. Scores were given by two blinded investigators within a 4 min interval. During the first 7 dpi (days post-injury), mice were scored daily, followed by an analysis each second or third day. For the analysis, the mean of the left and right hind limb scores for each animal were used. Mice were excluded from the experiment in case they (1) did not show an increase in their BMS score (lesion too severe) or (2) had an average BMS score of 1.5 or higher 1 dpi (lesion too weak). 

### 2.5. Sample Collection and DNA Isolation

Fecal samples were collected at the following time points: 1 day before SCI and 28 dpi, using metabolic cages to prevent contamination. The pellets were transferred to sterile tubes, immediately frozen, and stored at −80 °C until further processing. DNA extraction was performed using a previously described protocol [51]. In brief, fecal samples were transferred to a 2 mL tube containing a 200 mg mixture of 0.1–0.5 mm glass beads and 1.5 mL of lysis buffer (ASL) (Qiagen, Antwerp, Belgium). Feces were mechanically disrupted using the bead-beating method. The manufacturer’s procedure was slightly adapted by prolonging the proteinase K incubation time to 2 h at 70 °C. Distilled water was used as a negative control. The extracted DNA was stored at −20 °C until further use.

### 2.6. 16S rRNA Gene Amplification 

Amplification of the V4 region (F515/R806) of the 16S ribosomal (r)RNA gene was performed as previously described [52]. In brief, 25 ng DNA was used per PCR reaction for DNA-based amplicon sequencing. The following PCR conditions were utilized: (1) initial denaturation of 30 s at 98 °C and (2) 25 cycles of 10 s at 98 °C, 20 s at 55 °C, and 20 s at 72 °C. Each sample was amplified in triplicate and pooled afterwards. Following normalization, PCR amplicons were sequenced on an Illumina MiSeq platform PE300 (Illumina, Inc., San Diego, CA, USA).

### 2.7. Analysis and Processing of 16S rRNA Gene Sequencing Data

16S rRNA amplicons of region V4 were bioinformatically processed using QIIME2 [53]. The DADA2 plugin was used for quality checking (default parameters), reads trimming, and clustering into Operational Taxonomic Units (OTUs) [54]. OTUs are then assigned taxonomically using the VSEARCH algorithm (https://github.com/torognes/vsearch; accessed on 6 April 2023) and the Silva database v128 (https://www.arb-silva.de/; accessed on 6 April 2023). To normalize the gut microbiota composition, rarefaction was performed at 9962 reads of depth. Alpha diversity was assessed using Richness and Shannon metrics, while the beta diversity dissimilarity matrix was computed with the Bray–Curtis dissimilarity method. Both alpha and beta diversity indexes were calculated with the “vegan” package in R Version 2.5-7. All statistical analyses were conducted using the R software (https://www.R-project.org/; accessed on 24 May 2023; Version 4.1.0). Beta diversity indexes were visualized as Principal Coordinates Analysis (PCoA) generated using the “vegan” package. For PCoAs, data separations were tested via permutation test with pseudo-F ratios (“adonis” function from the “vegan” package). To evaluate the contribution of bacteria in the overall composition among groups, a preliminary analysis with the Kruskal–Wallis test was performed, and bacteria of interest were then further checked for each comparison of interest using the Wilcoxon test. To predict gut metagenomic functions, we performed functional annotation of representative sequences using PICRUSt 2, a bioinformatics software package to predict metagenome functional content from 16S rRNA gene sequencing data (Version 2.4.1). For the PICRUSt 2 pipeline, the raw count data were imported in the Python programming environment and run through the PICRUSt2 pipeline with default parameters. Finally, the differences between groups were statistically compared in R software (https://www.R-project.org/; accessed on 24 May 2023; Version 4.2.0) using Wilcoxon test and Kruskal–Wallis test functions and *p* values adjusted by the Benjamini–Hochberg method. A false discovery rate (FDR) ≤ 0.05 was considered statistically significant: * *p* ≤ 0.05; ** *p* ≤ 0.01; *** *p* ≤ 0.001. 

### 2.8. Statistics 

Data were analyzed using GraphPad Prism version 9.4.1 (GraphPad Software). Normal distribution was calculated by Shapiro–Wilk normality test with a significance level of 0.05. Functional recovery was calculated by unpaired t-test for normally distributed data. Data are presented as mean ± standard error of the mean (SEM). Differences with *p* values ≤ 0.05 were considered significant.

## 3. Results

### 3.1. Hemisection SCI Induces a Significant Change in Gut Microbiota Composition 

**Figure 1:**
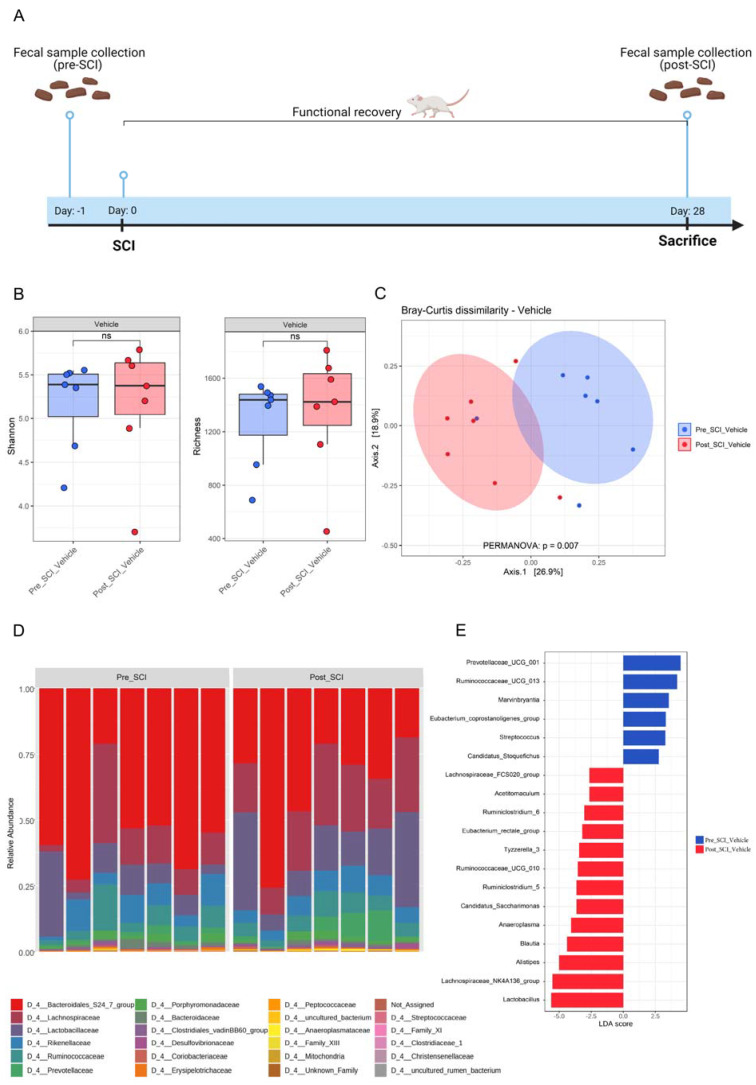
Impact of spinal cord injury on the composition and richness of the gut microbiota. (**A**) Schematic representation of the experimental setup. Fecal samples were collected 1 day before spinal cord injury (SCI) induction. From day 1 post-injury, functional recovery was assessed using the Basso Mouse Scale (BMS) score. After 28 days, mice were sacrificed, and fecal samples were collected again. n = 7. (**B**) Effect of SCI on alpha diversity of gut microbiota in fecal samples of vehicle-treated mice, Shannon (left) and microbial richness (Observed OTUs) (right). (**C**) The impact of SCI on the overall composition of the gut microbiota as Principal Coordinate Analysis of the Bray–Curtis dissimilarity matrix between samples from the vehicle-treated group. The distance between centroids of the two groups was tested using the PERMANOVA test (function “adonis” in the R package “vegan”). (**D**) Stacked bar graph of relative abundances (%) for individual gut microbiota composition at family level pre- and post-SCI. (**E**) The main bacteria contributing to the differences in composition before and after SCI in the vehicle-treated group. Linear Discriminant Analysis (LDA) effect size analysis (LEfSe) revealed significant bacterial differences in fecal microbiota pre- and post-SCI.

A large majority of the comorbidities that develop after SCI are linked to alteration of the gut microbiota. All preclinical studies investigating these changes were conducted in either contusion or compression animal models which are characterized by uninjured fibers that may lead to artifacts [47]. Therefore, we used a hemisection injury model in which no axonal preservation occurs. We investigated the composition of the gut microbiome before and after SCI in BALB/cJRj mice. Fecal samples were collected 1 day prior and 28 days after SCI and prepared for bacterial 16S rRNA gene sequencing (Figure 1A). The total number of filtered sequences in the samples collected from the animal groups before and after SCI was 1,034,473, and the average sequencing coverage was 32,013 sequences per sample with minimum and maximum coverages of 9962 and 45,152 reads, respectively. Based on the alpha diversity indices, there were no obvious changes in Shannon and bacterial community richness between the pre- and post-SCI groups (Figure 1B). The beta diversity of the gut microbiota was assessed using PCoA based on the Bray–Curtis distance matrix at the OTU level (Figure 1C). In contrast to the alpha diversity, PERMANOVA analysis for beta diversity and community composition showed that there were significant differences in fecal microbial communities between the two groups (PERMANOVA, R2 = 0.2, *p* = 0.007) (Figure 1C). These results indicate that SCI indeed has an influence on bacterial community composition of the gut. 

At the phylum level, the fecal microbiota collected from the pre-SCI group was dominated by Bacteroidetes (65.1%), followed by Firmicutes (33.9%), Proteobacteria (0.42%), Actinobacteria (0.24%) and Tenericutes (0.15%). In contrast, the post-SCI mice showed the microbiota dominated by Firmicutes (50%) followed by Bacteroidetes (48.3%), Proteobacteria (0.66%), Tenericutes (0.37%) and Actinobacteria (0.33%) (Figure S1A). 

Despite the change in the mean relative abundance of Bacteroidetes and Firmicutes, we observed no significant differences in the Firmicutes/Bacteroidetes ratio between pre- and post-SCI mice (Figure S1B). Taxonomic classification of bacterial families revealed that the most dominant family in fecal samples of pre-SCI mice was Muribaculaceae (53.1%), followed by Lachnospiraceae (14.58%), Lactobacillaceae (10.76%), Ruminococcaceae (7.4%) and Rikenellaceae (7.24%) (Figure 1D and Figure S1C). In contrast, post-SCI mice revealed a lower abundance of Bacteroidales S24-7 group (35.73%) and a higher presence of Lachnospiraceae (23.62%) and Lactobacillaceae (18.84%) (Figure 1D and Figure S1C). To identify additional high-dimensional biomarkers of gut microbiota in the pre- and post-SCI groups, we applied the LDA effect size algorithm (LEfSe) to identify differentially abundant bacterial taxa in both groups. LEfSe analysis revealed that six taxa were increased while 13 phylotypes were decreased in the mice before SCI (Figure 1E). Prevotellaceae UCG-001, Ruminococcaceae UCG-013, and Marvinbryantia showed a higher LDA score based on LEfSe, reflecting a significant increase in abundance in the mice before SCI compared with the mice after SCI. In contrast, *Lactobacillus*, the Lachnospiraceae NK4A136 group, *Alistipes*, and *Blautia* were enriched in the group of mice after SCI (Figure 1E). Our results show that SCI leads to changes in the beta diversity of the microbiome without affecting the alpha diversity of the gut bacteria. These changes are reflected by a significant separation of the gut microbiota between pre- and post-SCI mice groups, suggesting that hemisection SCI significantly influences the microbial community of the gut.

### 3.2. Anti-Inflammatory rIL-13 Treatment Improves Functional Recovery and Counteracts SCI-Induced Gut Dysbiosis in Mice 

**Figure 2:**
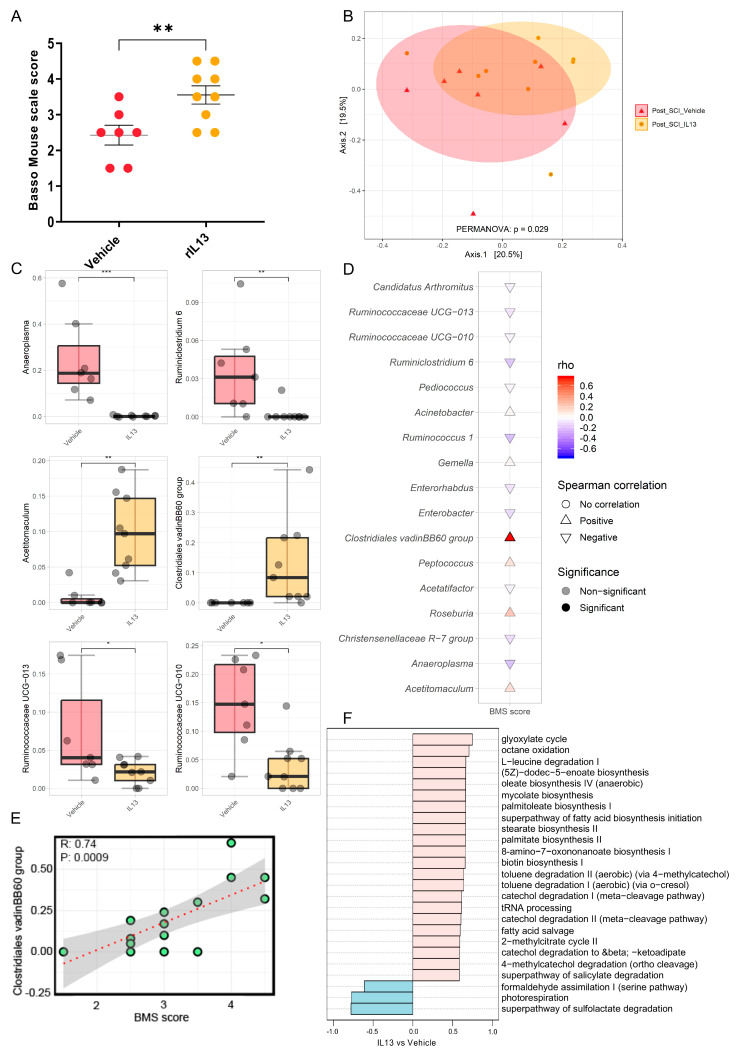
Effect of recombinant IL-13 treatment on functional recovery and gut microbiota after spinal cord injury. Starting 1 h after spinal cord injury (SCI) induction, BALB/cJRj mice received vehicle (PBS) or recombinant (r)IL-13. (**A**) At 28 dpi, rIL-13-treated mice showed significantly improved functional recovery compared to the vehicle group. Data are expressed as mean ± SEM. n = 7–9 mice/group. One independent experiment. (**B**) The effect of rIL-13 treatment on the composition of the gut microbiota of SCI mice as Principal Coordinate Analysis of the Bray–Curtis dissimilarity matrix between samples from both groups after treatment (post-SCI). The distance between the centroids of the two groups was tested using the PERMANOVA test (function “adonis” in the R package “vegan”). (**C**) Relative abundance of major genera affected by treatment (by time point for both vehicle and rIL-13 groups) presented as a boxplot; statistical comparisons between groups were performed using the Wilcoxon test. (**D**) Cuneiform plot for Spearman’s rank correlation between BMS values and relative abundance of bacterial genera for all samples (vehicle- and rIL-13-treated group). (**E**) Spearman’s rank correlation between the relative abundance of Clostridiales vadinBB60 group and BMS in all samples after a specific time point of the cohort (post-SCI vehicle and post-SCI rIL-13). (**F**) Spearman’s rank correlation between BMS and gut predictive metagenomic functions at a later time point in the cohort (post-SCI vehicle and post-SCI rIL-13). PICRUSt2 output is shown as a bar graph for the KEGG orthology annotation. *p* values of (**C**,**D**) are adjusted using the FDR method. * *p* ≤ 0.05; ** *p* ≤ 0.01; *** *p* ≤ 0.001.

Il-13 has been described to exert anti-inflammatory effects in SCI models [43]. Therefore, we evaluated whether systemic rIL-13 treatment could influence functional recovery after SCI and whether this relates to the gut microbiota composition (Figure S2A). Our data showed that rIL-13 treatment significantly improved functional recovery compared to vehicle at 28 dpi (Figure 2A). Of note, rIL-13-treated mice reached an average score of 3.6, which is more than 1.1 points higher than the vehicle group with a mean score of 2.4. Overall, this means that highly impaired mice that show only extensive ankle movement develop an occasional plantar-stepping pattern [50]. We further investigated the gut microbiota differences between the rIL-13-treated and control groups. Analysis of alpha diversity of the gut microbiome using the Shannon and richness indices showed that the difference did not reach statistical significance (Figure S2B). However, PERMANOVA analysis revealed that bacterial composition significantly differed (PERMANOVA, R2 = 0.11, *p* = 0.029) between the two groups (Figure 2B). Next, we examined the differences in microbial community composition between the rIL-13- and vehicle-treated SCI mice. The phylogenetic classification of bacterial phyla revealed a higher Firmicutes/Bacteroidetes ratio in the rIL-13-treated mice group, although the difference did not reach statistical significance (Figure S2C,D). Interestingly, at the genus level, we found significant highlighted differences in the abundance of certain microbial taxa between the two groups in the gut (Figure 2C). We observed that the presence of Clostridiales vadin BB60 and *Acetitomaculum* were increased in the rIL-13-treated group, while *Anaeroplasma*, *Ruminiclostridium_6* and Ruminococcaceae UCG-013 and Ruminococcaceae UCG-010 were decreased in relative abundance following rIL-13 treatment compared to vehicle. Spearman’s correlations were calculated between the functional recovery of the mice after SCI. Interestingly, Clostridiales vadin BB60 had a significant positive correlation (r = 0.74, *p* = 0.0009) with functional recovery of mice after SCI regardless of treatment groups (Figure 2D,E). 

To determine the functional enrichment of gut microbiota in rIL-13-treated mice, functional assessment with PICRUSt was performed to predict the composition of KEGG pathways of microbiota structure. Correlation analysis showed that genes related to the glyoxylate cycle, octane oxidation, mycolate biosynthesis, and palmitoleate biosynthesis I were the predominant gene family in all of rIL-13-treated mice (Figure 2F), whereas the sulfolactate degradation superpathway and formaldehyde assimilation I were enriched in the control group (Figure 2F). Overall, these data show that rIL-13 treatment not only improved functional recovery but also induced significant shifts in the gut microbiota composition with potentially anti-inflammatory features. 

## 4. Discussion

The gut microbiome has been shown to act as a pivotal player in SCI pathophysiology. Changes in homeostatic microbiota composition as a result of the disrupted autonomic nervous system following SCIs have been shown to limit functional outcomes [47]. To date, most studies on changes in the microbiota after SCI have been performed in contusion and compression injury models characterized by uninjured fibers that may lead to artifacts, and information on changes in hemisection injury models without fiber preservation is lacking [47]. In the current study, we thus characterized the changes in gut microbiome composition after hemisection SCI. In addition, we aimed to investigate the effects of anti-inflammatory rIL-13 treatment on microbiome imbalance and locomotion to identify changes in the gut microbiome that correlate with functional recovery after SCI. 

Consistent with recent research suggesting an association between SCI and changes in gut microbiota in C57BL/6 mice [31,39,55], our results showed that the composition of the gut microbiome was also significantly altered in BALB/cJRj mice after SCI. However, we did not detect profound changes in alpha diversity between groups. In accordance with previous results indicating that the relative abundance of Bacteroidetes decreased significantly over time after SCI in C57BL/6 mice [39,56], we found a lower abundance of Bacteroidetes and a higher prevalence of Firmicutes in BALB/cJRj mice after SCI. However, no differences in the Firmicutes/Bacteroidetes ratio between mice before and after SCI were observed. Previous studies investigated the effects of SCI on the gut microbiota of C57BL/6 mice and found changes in the abundance of certain bacterial taxa, including decreases in *Lactobacillus*, Lachnospiraceae_NK4A136, and *Blautia* [39,55,57]. In contrast, our results showed that SCI significantly increased the relative abundance of *Lactobacillus*, Lachnospiraceae_NK4A136, *Blautia*, and *Alistipes* in the BALB/cJRj mouse model. These differences in bacterial abundance could be due to several factors, including differences between animal strains, the severity of injury, housing conditions, and further individual variations like extraction protocols and time points of sampling. 

Interestingly, Kigerl et al. showed that preventing intestinal dysbiosis by prophylactic probiotic treatment improved locomotion after SCI [39]. This was associated with an increase in the number of CD4^+^CD25^+^FoxP3^+^ Tregs and CD11c^+^ DCs, indicating the induction of systemic anti-inflammatory effects. One possible factor linked to enhanced anti-inflammatory activity by increases of Tregs and DCs with immunomodulatory properties is IL-13 [58]. In addition, SCI-induced changes in the gut lead to a decrease in butyrate-producing commensals, which contributes to the induction of pro-inflammatory macrophages [41]. We have previously demonstrated the importance of IL-13 in polarizing macrophages toward an anti-inflammatory phenotype [43,44]. In line with this, we have further shown that local administration of IL-13 using macrophage-based delivery improves functional recovery after SCI [43,44]. In this study, we examined whether systemic rIL-13 treatment had effects on microbiome composition and locomotion. Notably, in line with significantly improved functional recovery of mice receiving rIL-13, the analysis of the microbiota showed significant differences between the rIL-13 and vehicle groups, with particular increases in the abundance of Clostridiales vadin BB60 and *Acetitomaculum* in the rIL-13 group.

Interestingly, the prediction of metabolic pathways based on the microbial 16S ecosystem in rIL-13-treated mice compared to controls indicated that palmitoleate biosynthesis was significantly activated. Palmitoleic acid has been shown to have anti-inflammatory properties by inhibiting NFκB pathway, independently of PPARs in macrophages [59]. Another report suggested that the anti-inflammatory effects of palmitoleic acid could be due to a reduction in TLR4-dependent and TNF-α-independent signaling [60]. Deficiency of IL-13 has further been described as being correlated with alterations of the gut microbiome and decreases in SCFA production in mice. These alterations were associated with increased susceptibility to colitis and impaired gut barrier function, while administration of IL-13 restored SCFA levels and reduced colitis symptoms in IL-13-deficient mice [61]. It thus would be of interest to investigate in future studies in SCI or other (auto)immune pathologies whether the manipulation of the gut microbiome by inducing/reestablishing an IL-13-prone microbiome may directly have the potential to exert similar effects in enhancing anti-inflammatory immunomodulation and regeneration.

In summary, our data demonstrate that gut dysbiosis also occurs in a hemisection SCI model. In addition, rIL-13 treatment improved functional outcomes at 28 dpi after SCI and was also accompanied by distinct changes in the gut microbiota, particularly by alterations in the abundance of Clostridiales vadin BB60. PICRUSt pathway analysis further pointed towards the induction of anti-inflammatory features observed in the gut microbiota of rIL-13-treated mice. However, whether changes in the microbiota following rIL-13 treatment could directly contribute to improved functional recovery and how it may relate to the immune status remains to be validated in future studies. Notwithstanding, our results could have important implications for developing novel treatment options for SCI. 

## Data Availability

The data sets used and/or analyzed during this study are available from the corresponding authors upon reasonable request.

## Supplementary Materials

The following supporting information can be downloaded at: https://www.mdpi.com/article/10.3390/nu15194184/s1, Figure S1: Effects of SCI on phyla and families of the gut microbiota. (A) Stacked bar graph of group means of major phyla detected in fecal samples from mice pre- and post-spinal cord injury (SCI) in the vehicle treated group. (B) Firmicutes/Bacteroidetes ratio in fecal samples from the vehicle group before and after SCI; statistical comparisons between groups were performed using the Wilcoxon test. (C) Stacked bar graph of group means of major families detected in fecal samples from mice before and after SCI in the vehicle treated group. Figure S2: Effect of rIL-13 treatment on the gut microbiota of SCI mice. (A) Schematic representation of the experimental design. Fecal samples were collected 1 day before induction of spinal cord injury (SCI). The next day, SCI surgery was performed, and after 1 hour, mice received the first treatment with either vehicle or recombinant interleukin-13 (rIL-13), which was continued for the next 6 consecutive days. From day 1 post-injury, functional recovery was assessed using the Basso Mouse Scale (BMS). After 28 days, mice were sacrificed and fecal samples were collected again. (B) Effect of rIL-13 treatment on the alpha diversity of the SCI-derived gut microbiota in fecal samples, Shannon (left), and microbial richness (observed OTUs) (right); statistical comparisons between groups were performed using the Wilcoxon test. (C) Stacked bar graph of group means of major phyla detected in mouse fecal samples by time point of SCI-derived gut microbiota in the vehicle and rIL-13 treated groups. (D) Firmicutes/Bacteroidetes ratio in the vehicle and rIL-13 treated samples after 28 days post-injury; statistical comparisons between groups were performed using the Wilcoxon test. Table S1: Acronyms and Abbreviations.
