# Supplementary material for: Effects of Recombinant IL-13 Treatment on Gut Microbiota Composition and Functional Recovery after Hemisection Spinal Cord Injury in Mice
Source: Nutrients. 2023 Sep 27;15(19):4184. doi: 10.3390/nu15194184 (PMC10574124; doi:10.3390/nu15194184)
Supplement: Supplementary file 1 [file nutrients-15-04184-s001.zip › nutrients-2543948-supplementary.pdf]

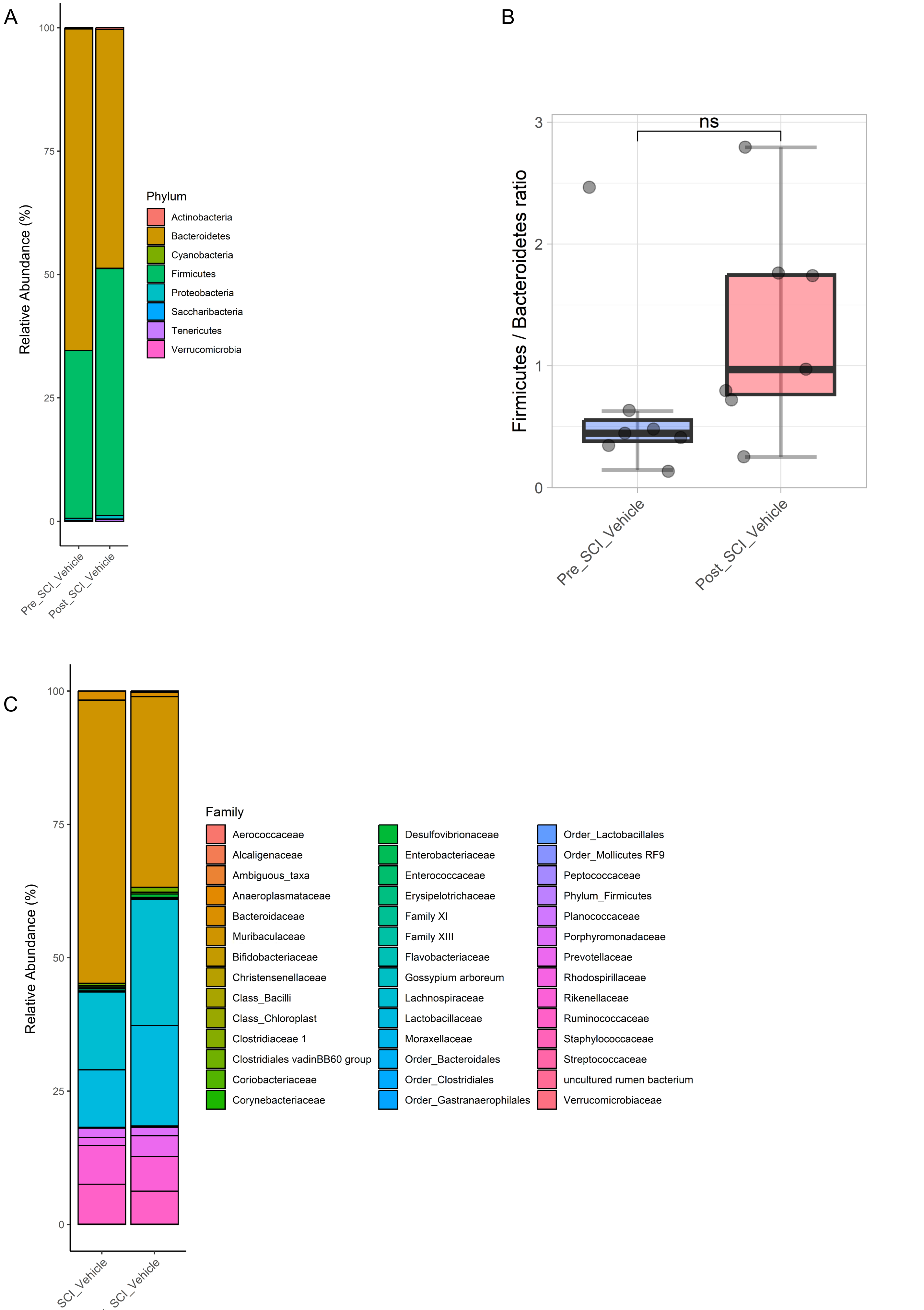
A
B
C
Relative Abundance (%)
100
75
50
25
0
Pre_SCI_Vehicle
Post_SCI_Vehicle
Phylum
Actinobacteria
Bacteroidetes
Cyanobacteria
Firmicutes
Proteobacteria
Saccharibacteria
Tenericutes
Verrucomicrobia
Firmicutes / Bacteroidetes ratio
ns
3
2
1
0
Family
Aerococcaceae
Alcaligenaceae
Ambiguous_taxa
Anaeroplasmataceae
Bacteroidaceae
Muribaculaceae
Bifidobacteriaceae
Christensenellaceae
Class_Bacilli
Class_Chloroplast
Clostridiaceae 1
Clostridiales vadinBB60 group
Coriobacteriaceae
Corynebacteriaceae
Desulfovibrionaceae
Enterobacteriaceae
Enterococcaceae
Erysipelotrichaceae
Family XI
Family XIII
Flavobacteriaceae
Gossypium arboreum
Lachnospiraceae
Lactobacillaceae
Moraxellaceae
Order_Bacteroidales
Order_Clostridiales
Order_Gastranaerophilales
Order_Lactobacillales
Order_Mollicutes RF9
Peptococcaceae
Phylum_Firmicutes
Planococcaceae
Porphyromonadaceae
Prevotellaceae
Rhodospirillaceae
Rikenellaceae
Ruminococcaceae
Staphylococcaceae
Streptococcaceae
uncultured rumen bacterium
Verrucomicrobiaceae

Figure S1

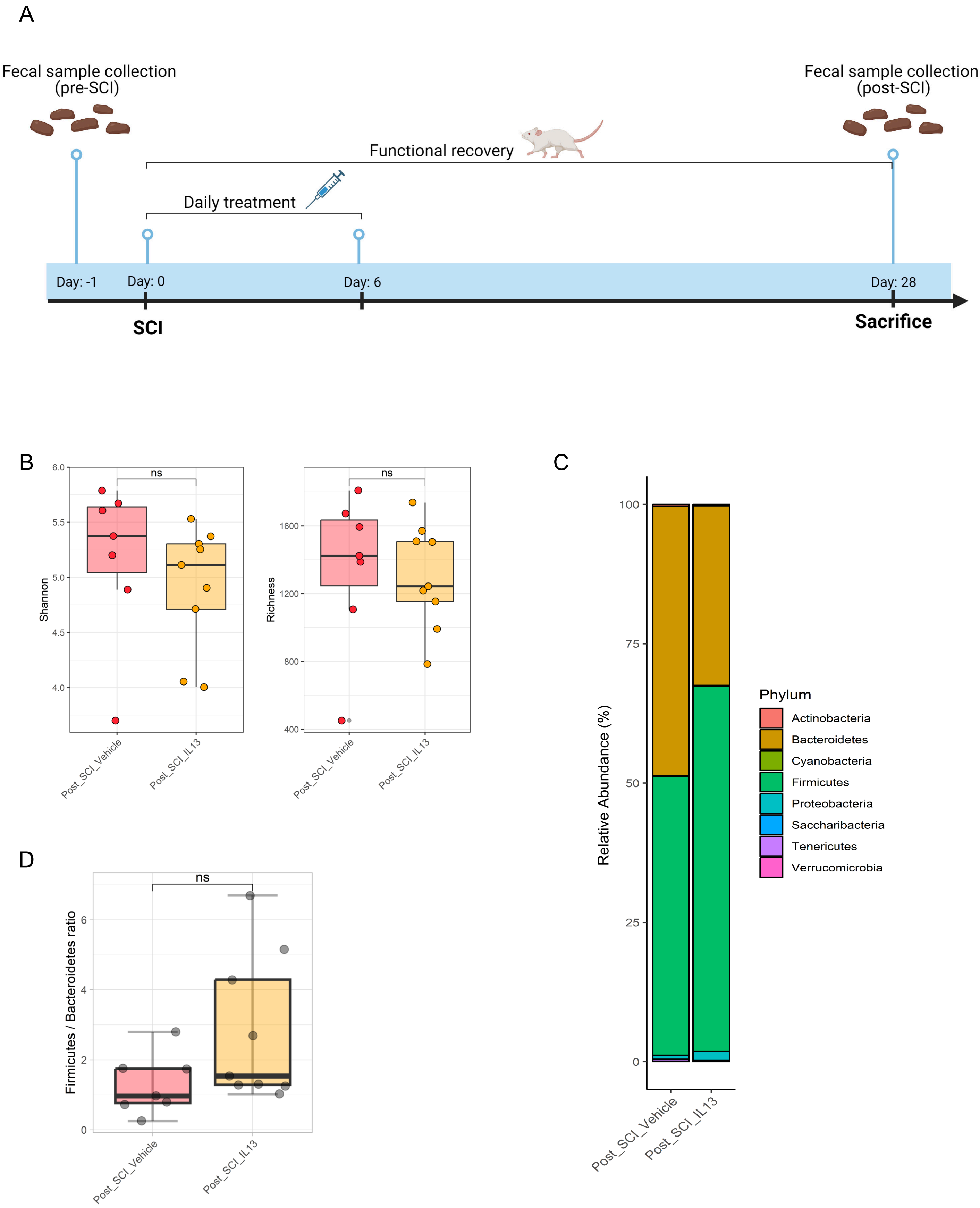
A
Fecal sample collection (pre-SCI)
Fecal sample collection (post-SCI)
Functional recovery
Daily treatment
Day: -1
Day: 0
Day: 6
Day: 28
SCI
Sacrifice
B
ns
Shannon
Richness
Post_SCI_Vehicle
Post_SCI_IL13
C
Relative Abundance (%)
Phylum
Actinobacteria
Bacteroidetes
Cyanobacteria
Firmicutes
Proteobacteria
Saccharibacteria
Tenericutes
Verrucomicrobia
D
Firmicutes / Bacteroidetes ratio

Figure S2

Table S1. Acronyms and Abbreviations

Abbreviation	Definition
ASD	Autism Spectrum Disorder
BMS	Basso Mouse Scale
CNS	Central Nervous System
FDR	False Discovery Rate
GI	Gastrointestinal
LDA	Linear Discriminant Analysis
LEfSe	Linear Discriminant Analysis Effect Size
MS	Multiple Sclerosis
NFκB	Nuclear factor kappa-light-chain-enhancer of activated B cells
OTU	Operational Taxonomic Unit
PBS	Phosphate Buffered Saline
PPARs	Peroxisome Proliferator-Activated Receptors
rIL-13	Recombinant Interleukin-13
rRNA	Ribosomal Ribonucleic Acid
SCFA	Short-Chain Fatty Acid
SCI	Spinal Cord Injury
SEM	Standard Error of the Mean
TLR4	Toll-Like Receptor 4
TNF-α	Tumor Necrosis Factor Alpha
WT	Wild Type
